# Comparative Analysis of Gut Microbiota in Captive and Wild Oriental White Storks: Implications for Conservation Biology

**DOI:** 10.3389/fmicb.2021.649466

**Published:** 2021-03-25

**Authors:** Hong Wu, Fang-Ting Wu, Qi-Hai Zhou, Da-Peng Zhao

**Affiliations:** ^1^Tianjin Key Laboratory of Conservation and Utilization of Animal Diversity, College of Life Sciences, Tianjin Normal University, Tianjin, China; ^2^Key Laboratory of Ecology of Rare and Endangered Species and Environmental Protection, Ministry of Education, Guangxi Key Laboratory of Rare and Endangered Animal Ecology, Guangxi Normal University, Guilin, China

**Keywords:** gut microbiota, oriental white storks, microbial diversity, high-throughput sequencing, conservation

## Abstract

The oriental white stork (*Ciconia boyciana*) is considered an endangered species based on the International Union for Conservation of Nature (IUCN) Red List. This study presents the first evidence on comparative analysis of gut microbial diversity of *C. boyciana* from various breeding conditions. To determine the species composition and community structure of the gut microbiota, 24 fecal samples from Tianjin Zoo and Tianjin Qilihai Wetland were characterized by sequencing 16S rRNA gene amplicons using the Illumina MiSeq platform. Firmicutes was found to be the predominant phylum. Analysis of community structure revealed significant differences in the species diversity and richness between the populations of the two breeding conditions. The greatest α-diversity was found in wild *C. boyciana*, while artificial breeding storks from Tianjin Zoo had the least α-diversity. Principal coordinates analysis showed that the microbial communities were different between the two studied groups. In conclusion, this study reveals the species composition and structure of the gut microbiota of oriental white storks under two breeding conditions, and our findings could contribute to the integrative conservation of this endangered bird.

## Introduction

Gut microbiota plays a crucial role in the health of animal hosts through many factors, such as genetics, geography, nutrition, diet, and immunity (Kohl, 2012; van Dongen et al., 2013; Kong et al., 2014; Waite and Taylor, 2014, 2015). It is well-established that the gut microbiota of an animal functions from the moment the animal is born, the amount and species of gut microbiota are not fixed (Xiong et al., 2019; Li et al., 2020). The external environment and the host itself influence the diversity of gut microorganisms (Ley et al., 2006; Mueller et al., 2006; Waite and Taylor, 2014). Thus, research on gut microbiomes can improve survival of animals in different living environments and an emerging suite of novel tools from this research is being applied for animal health and conservation field (Clemente et al., 2012; Lee and Hase, 2014; Waite and Taylor, 2015).

Studies of gut flora are being conducted in many animals, but among these, there is considerably less research on birds than mammals (Clemente et al., 2012). Many factors influence the intestinal flora of birds, such as population diversity and different life-history characteristics including migratory behavior, diet, flight ability, and mating system (Xenoulis et al., 2010; van Dongen et al., 2013; Stanley et al., 2015; Colston and Jackson, 2016).

Nowadays 16S rRNA high-throughput sequencing technologies has been used to determine composition and compare structures of the intestinal flora recent years (Cole et al., 2009; Wang et al., 2015; Wu et al., 2016; Cui et al., 2019). With the development of high-throughput sequencing, the study of avian gut microbiota has become feasible and economical (Salter et al., 2014; Hird et al., 2015). Consequently, the amount of data generated in this field has markedly increased, providing more information on the relationships among gut microbiomes across different bird species and habitats (Michel et al., 2018). For instance, it is found that cecal microbes of wild and captive western grouse are significantly different since the living environments, including diet and exercise intensity, differ between the two groups (Wienemann et al., 2011). Wang et al. (2020) compared the compositions and differences in gut microbiomes of black-necked cranes (*Grus nigricollis*) from six wintering areas in China, and found that the overwintering food sources might lead to specific gut microbes (Wang et al., 2020).

The oriental white stork (*Ciconia boyciana*) is a large water fowl that is endangered due to the loss of habitat and disturbance through human activities (Cheng et al., 2019; Fan et al., 2020). The main breeding areas of oriental white storks are limited to northern China and southeastern Russia (Liu et al., 2008; Zan et al., 2008; Yamada et al., 2019). The number of wild oriental white storks has declined sharply past decade, with approximately 1,000–2,499 mature individuals globally (IUCN, 2020), and this species is listed as nationally protected animals in China (Itoh et al., 1997; Cardoso et al., 2016; Han et al., 2016). Conservation of *C. boyciana* is of great significance and considerable efforts from various research fields have been conducted in this endangered species (e.g., Liu et al., 2008; Han et al., 2016; Wei et al., 2016; Cheng et al., 2020). However, the environmental influence on the intestinal flora of oriental white storks have yet to be elucidated. Considering the integrative conservation of endangered species, one related comparative analysis is therefore required to understand whether gut microbiota of oriental white storks differ between different regions.

The present study aimed to compare fecal microbial community compositions and diversity of oriental white storks from two breeding conditions by using the Illumina MiSeq platform for high-throughput sequencing for the first time. Differences in the community structure as well as species composition of the fecal microbiota in *C. boyciana* were compared, and the unique and shared bacteria were identified with the aim of finding relationships between different groups. Results of the study will facilitate understanding of the fecal microbiota in oriental white storks under two breeding conditions and could help improve population conservation of this endangered species.

## Materials and Methods

### Ethics Statement

All applicable international, national, and/or institutional guidelines for the care and use of animals were strictly followed. All animal sample collection protocols complied with the current laws of China. According to the Law of the People’s Republic of China on the Protection of Wildlife (Order of the President of the People’s Republic of China, No. 16, 2018), all experimental procedures carried out in this study were under permitted. The non-invasive genetic sampling taken in this work enables collecting samples (fecal samples) without the need to directly disturb or contact individuals of oriental white storks.

### Sample Collection

Twelve fecal samples of oriental white storks (*Ciconia boyciana*) were collected at Tianjin Zoo (Z Group) and Tianjin Qilihai Wetland (W group), respectively in 2019 (Table 1). Tianjin Qilihai Wetland is one of the main stopover habitats of wild *C. boyciana* during their migration periods. All samples were carefully taken from the inside of the fecal matter using sterilized equipment, and then were placed on dry ice and transported to the laboratory as soon as possible.

**TABLE 1:** Basic information on experimental samples.

Sample group	Collection location	Collection time	Sex composition	Age composition
**Z group**	Tianjin Zoo	2019/10/31, 2019/11/20	8 males and 4 females	The age ranges from 4 years old to 30 years old, with an average age of 15 years old
**W group**	Tianjin Qilihai Wetland	2019/12/3	unknown	unknown

### DNA Isolation and Illumina MiSeq Sequencing

The total genomic DNA of all fecal samples was isolated by CTAB extraction method; Sodium dodecyl sulfate (SDS) extraction method; Guanidine thiocyanate (GuSCN) extraction method and TIANamp Stool DNA Kit (TIANGEN, China). For all methods, DNA extraction was conducted according to the references and manufacturer’s instructions (Hosomi et al., 2017; Kachiprath et al., 2018). The concentration and quality of DNA samples were determined by a NanoDrop 2000 spectrophotometer (Thermo Fisher Scientific, Waltham, MA, United States).

The V3–V4 region of the 16S rRNA gene was amplified based on the literature using specific PCR primers (Wu et al., 2016). Forward primer 338F (5′-ACTCCTACGGGAGGCAGCAG-3′) and reverse primer 806R (5′- GGACTACHVGGGTWTCTAAT-3′) were used (Caporaso et al., 2012). Amplification was carried out in 50-μL reactions with 10 ng template DNA and 25 μL Pfu DNA polymerase mix. The PCR cycle comprised an initial denaturation at 94°C for 5 min, followed by 30 cycles of 94°C for 15 s, 60°C for 15 s, and 72°C for 30 s.

Purified PCR amplicons were sequenced on an Illumina MiSeq platform at Majorbio Bio-Pharm Technology Co., Ltd., Shanghai, China. PE amplicon libraries were constructed, and all raw reads were screened according to the primer sequences.

### Bioinformatics, Sequence Analysis and Statistical Analyses

Raw files of the total genomic DNA from fecal samples were demultiplexed by QIIME (version 1.9.1), and high-quality reads were clustered as an operational taxonomic unit (OTU) by UPARSE pipeline (version 7.1) when the sets of sequences shared at least 97% identity (Edgar, 2013). In this study, gene sequence taxonomy was analyzed by RDP Classifier (version 2.2)^1^ (Wang et al., 2007) using a confidence threshold of 80% against the Silva 16S rRNA database (Release 128)^2^ (Quast et al., 2013).

Rarefaction curves were plotted for each sample to determine the abundance of communities and sequencing data of each fecal sample (Amato et al., 2013). α-diversity and species accumulation curve were calculated by mothur software (version v.1.30.1), which showed the complexity of gut flora species in two groups. β-diversities analysis was performed and principal coordinate analyses (PCoA) based on OTU compositions were determined, in order to investigate structural variation in microbial communities of two groups using unweighted and weighted UniFrac distance metrics (Lozupone and Knight, 2005). The one-way analysis of variance ANOVA (Permutational multivariate analysis of variance) was performed to compare α-diversity estimates and *p* < 0.05 was considered statistically significant. Differences in the UniFrac distances for pairwise comparisons were determined using the Student’s *t*-test.

Taxa abundances in two groups at the phylum, class, order, family, and genus levels were statistically compared by Metastats (an improved statistical method for analysis of metagenomic data) (White et al., 2009). Microbial functions were predicted by PICRUSt based on high-quality sequences and the linear discriminant analysis effect size (LEfSe) was used to present bacterial taxonomic distributions of sample communities (Segata et al., 2011).

## Results

### Comparison of Different DNA Extraction Methods

DNA extraction is a very critical step for high-throughput sequencing and the choice of a DNA extraction method has an important impact on DNA yield and purity. As the results shown, the GuSCN extraction method and the TIANamp Stool DNA Kit produced much higher DNA yields than the CTAB and SDS methods (Table 2). The obtained DNA yields by all the four extraction methods were higher than 10 ng/μl. The 260/230 absorbance ratios GuSCN extraction method and TIANamp Stool DNA Kit were above 1.8, indicating a low contamination with proteins. However, the average 260/280 absorbance ratios detected for the CTAB and SDS were below 1.8 which might suggest protein contamination from the two methods. In the study of oriental white stork’s fecal sample, the DNA obtained by GuSCN extraction method and TIANamp Stool DNA Kit could both be used successfully in library preparation for high-throughput sequencing. Moreover, DNA extraction using the GuSCN protocol was more cost efficient compared to the commercially TIANamp Stool DNA Kit.

**TABLE 2:** DNA yield and DNA purity (absorbance ratios of 260/280 nm) of four different DNA extraction methods.

	CTAB extraction	SDS extraction	GuSCN extraction	TIANamp Stool DNA Kit
DNA yield (ng/μl)	28.95 ± 2.96	16.89 ± 2.35	101.02 ± 1.45	98.12 ± 2.43
Abs 260 nm/280 nm	1.32 ± 0.58	1.53 ± 0.41	1.81 ± 0.35	1.79 ± 0.95

### Sequencing Data Analysis

Across the 24 samples of oriental white storks, a total of 1,263,339 raw reads with an average length of 415 bp were obtained after denoising by Illumina MiSeq sequencing. The total number of raw reads, base pairs, and the mean length of the reads were compared in all the 24 samples (Supplementary Table 1). The number of sequences varied from 42,700 to 70,383, and a total of 2,390 OTUs with 97% sequence similarity threshold representing 43 bacterial phyla were identified in the feces of *C. boyciana*. The α-diversity indices for bacteria, including Sobs, Shannon, Chao1, ACE, and Good’s coverage are shown in Table 3. The total number of OTUs in the W group (2,134) was much higher than that in the Z group (1,555), these values include 1,299 OTUs shared by both groups. There was a significant difference in α-diversity indices between Z group and W group (*p* > 0.05) (Supplementary Figure 1). Average Good’s coverage was 99.28 ± 1.57% (mean ± SD) around all 24 fecal samples, and thus the majority of the bacterial diversity in feces was obtained thereby ensuring accuracy of the analyses (Table 3).

**TABLE 3:** Estimated OTU richness and diversity indices for each fecal sample of oriental white storks.

Sample ID	Sobs	ACE	Chao1	Shannon	Simpson	Coverage (%)
Z1	582	660.8866	646.901	3.3475	0.098	0.9983
Z2	571	710.6577	685.8542	2.9449	0.1824	0.9968
Z3	245	285.3304	279.45	2.9651	0.1116	0.999
Z4	534	644.1314	619.6827	1.4346	0.6281	0.9974
Z5	279	415.1391	436.814	1.4611	0.3824	0.9974
Z6	208	456.6761	346	1.3184	0.3959	0.9983
Z7	1459	1764.5793	1744.6276	4.621	0.046	0.991
Z8	1284	1552.5374	1530.0135	3.8731	0.0896	0.9924
Z9	1580	1886.014	1860.5814	4.3103	0.0946	0.9926
Z10	1106	1180.2196	1211.6117	5.0927	0.0197	0.9967
Z11	1244	1411.9333	1384.2027	3.321	0.2654	0.9951
Z12	792	880.9801	888.6757	2.956	0.1936	0.9971
W1	2783	3584.8701	3612.0146	6.1687	0.0099	0.9805
W2	2664	3493.8137	3403.4703	5.606	0.0212	0.9805
W3	3000	3469.189	3424.9702	6.1289	0.0108	0.9877
W4	1091	2267.6478	1819.1977	3.6838	0.0633	0.9905
W5	2720	3713.3214	3695.4599	5.4219	0.0189	0.9848
W6	1604	2125.7942	2123.18	4.7485	0.0315	0.9893
W7	664	1397.0354	1014.0273	3.5062	0.0916	0.9937
W8	2406	3072.3858	3009.0795	5.7545	0.0114	0.9842
W9	1968	2459.6551	2446.1631	5.1929	0.0249	0.9873
W10	2958	3645.6935	3586	6.1753	0.0128	0.983
W11	2134	2353.736	2381.8266	5.8923	0.0106	0.9925
W12	2878	3662.8558	3666.8014	6.3666	0.0055	0.9833

To assess whether the depth of sequencing in this study was large enough to estimate the richness of gut microflora, the observed species rarefaction curves were calculated. According to species accumulation curves and Good’s coverage estimator, the curve tended to gradually flatten, indicating that the sample size collected was large enough to identify the majority of OTUs in the gut of oriental white storks (Supplementary Figure 2), and the high Good’s coverage indicated that the majority of bacterial phylotypes in all the samples were identified.

### Microbiota Composition and Relative Abundance of All Samples

**FIGURE 1:**
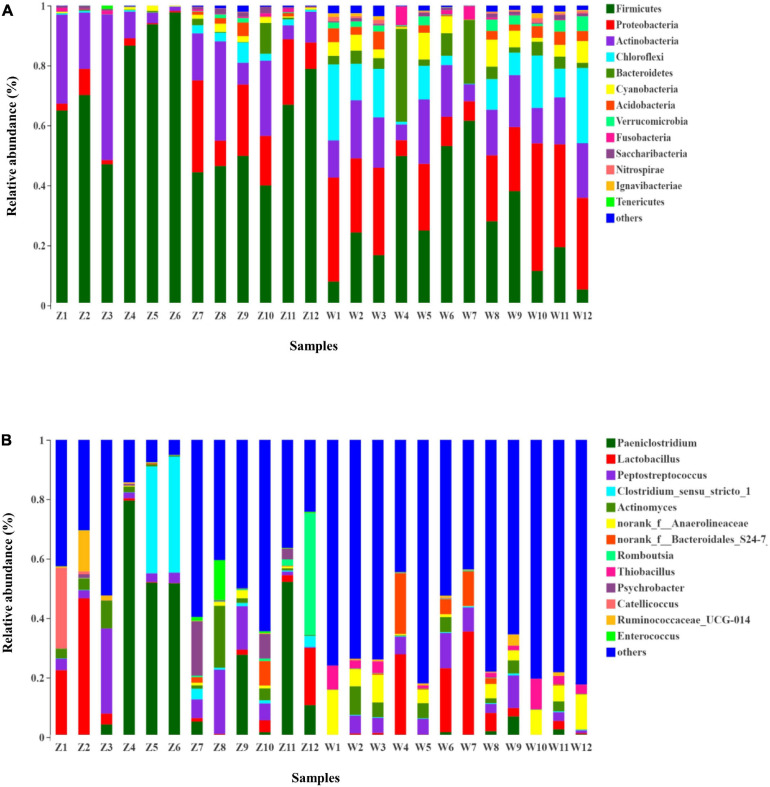
Microbial structure of all fecal samples at phylum and genus levels. **(A)** Bar-plots showing the abundance and distribution of the 11 most abundant phyla. **(B)** Bar-plots showing the abundance and distribution of the 11 most abundant genera.

The taxonomic composition of each fecal sample clustering at the 97% phylotype similarity level was outlined at phylum, class, order, family, and genus levels (Supplementary Table 2). Overall gut microbiomes of the oriental white storks comprised of 43 phyla, 96 classes, 188 orders, 338 families, 675 genera, and 1,222 species; microbial compositions at these different levels are shown in Figure 1 and Supplementary Figures 3–5, respectively.

At the phylum level, 43 prokaryotic phyla were identified from the 16S rRNA gene sequences (Figure 1A). Gut microbiota of *C. boyciana* from Z group was dominated by Firmicutes (65.59%), Actinobacteria (17.23%), and Proteobacteria (10.48%). These three dominant phyla accounted for 93.30% of all sequences across all the samples, while unclassified bacterial sequences only occupied 0.01% (Figure 1A and Supplementary Table 2). The gut microbiota from W group was dominated by Firmicutes (28.43%), Proteobacteria (23.58%), Actinobacteria (14.71%), Chloroflexi (11.57%), and unclassified bacterial sequences accounted for 0.04% of all sequences in the W group (Figure 1A and Supplementary Table 2).

**FIGURE 2:**
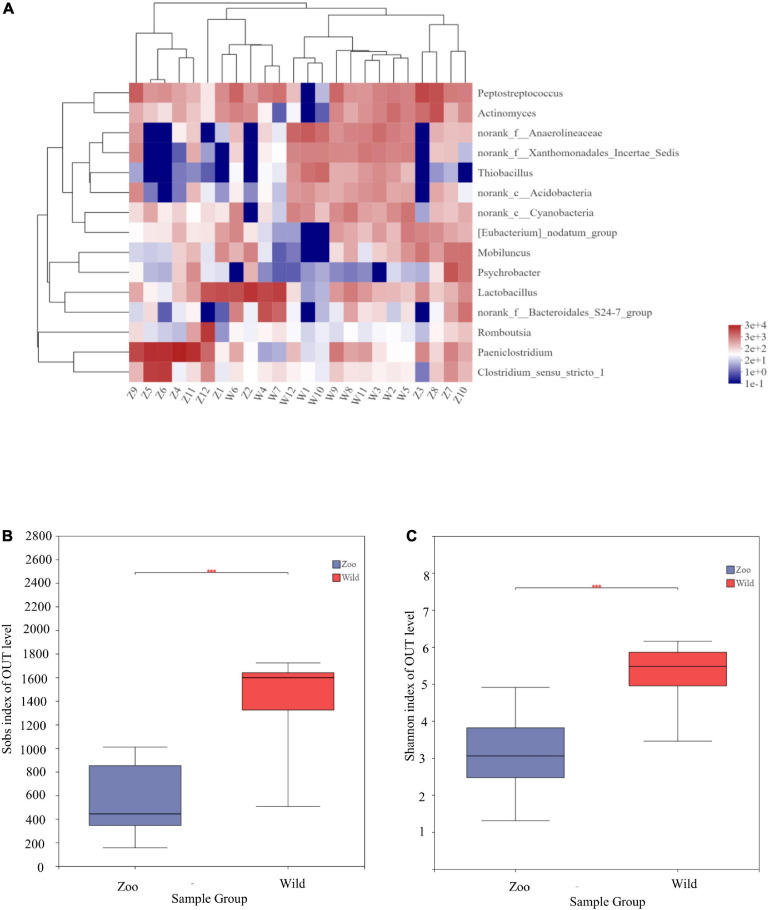
Community heatmap analysis at genus level and α-diversities of gut microbiota among the two populations. **(A)** Community heatmap analysis on genus level. **(B)** Bacterial community richness (measured by Sobs index) in the Z and W groups. **(C)** Bacterial community diversity (measured by Shannon index) in the Z and W groups. *p* < 0.05 was considered statistically significant. ***P ≤ 0.001.

At the genus level, 15 genera had abundances greater than 2% in all the sequences (Figure 1B). The most abundant genera in W group were *Lactobacillus* (8.33%), *norank_f_Anaerolineaceae* (5.92%), *norank_f_Xanthomonadales_Incertae_Sedis* (3.91%), *norank_f_Bacteroidales_S24-7_group* (3.38%), *norank_c_ Cyanobacteria* (3.35%), and *Thiobacillus* (2.943%). The most abundant genera in Z group were *Paeniclostridium* (23.82%), *Lactobacillus* (8.55%), *Peptostreptococcus* (7.78%), *Clostridium_sensu_stricto_1* (7.17%), *Actinomyces* (3.99%), and *Romboutsia* (3.77%). Mean relative abundances of the 10 most abundant genera are listed in Table 4. Community heatmap analysis at the genus level of the 24 samples can be seen in Figure 2A.

**TABLE 4:** Mean relative abundance of the 10 most abundant genera for each sample group.

Sample group	Genus (%)
Z group	*Paeniclostridium* (23.82)
	*Lactobacillus* (8.55)
	*Peptostreptococcus* (7.78)
	*Clostridium_sensu_stricto_1* (7.17)
	*Actinomyces* (3.99)
	*Romboutsia* (3.77)
	*Mobiluncus* (3.01)
	*Psychrobacter* (2.73)
	*Catellicoccus* (2.37)
	*Ruminococcaceae_UCG-014* (1.41)
W group	*Lactobacillus* (8.33)
	*norank_f_Anaerolineaceae* (5.92)
	*norank_f_Xanthomonadales_Incertae_Sedis* (3.91)
	*norank_f_Bacteroidales_S24-7_group* (3.38)
	*norank_c_Cyanobacteria* (3.35)
	*Thiobacillus* (2.94)
	*Actinomyces* (2.86)
	*Paenisporosarcina* (2.51)
	*norank_c_Acidobacteria* (1.92)
	*Planomicrobium* (1.68)

α-diversity, which refers to the diversity within a particular region, was calculated for two groups to examine differences between oriental white storks from Tianjin Zoo and Tianjin Qilihai Wetland. Community richness measured by OTUs (Sobs and Chao indices) showed that the index of the W group was higher than that of the Z group, indicating that richness of gut microbiota was greater in W group (Supplementary Figure 1). Differences between two groups were statistically significant (*p* < 0.05) (Figure 2B). Bacterial community diversity (Shannon index) was measured in the two groups, and the index of W group was higher than that of Z group (Supplementary Figure 1). There was a significant difference in community diversity between the W and Z groups (*p* < 0.05) (Figure 2C).

### β-Diversity Analysis and Community Structures

**FIGURE 3:**
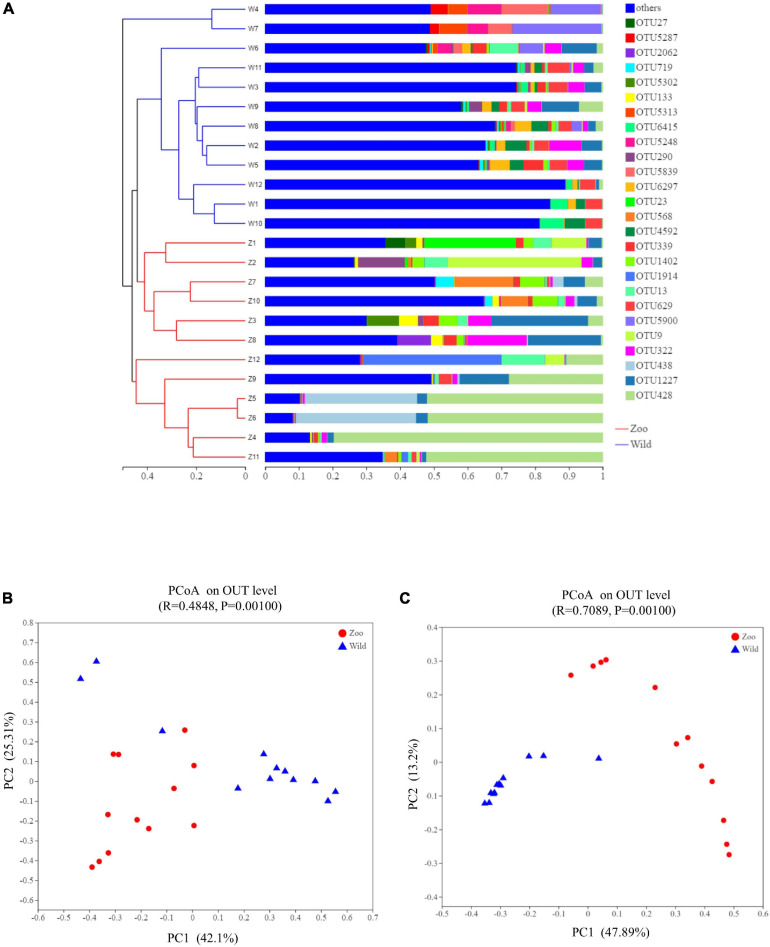
Clustering analysis of the gut microbiotas and PCoA plots analysis. **(A)** Clustering analysis of the evolution of gut microbiotas in the Z and W groups. Gut microbiota trees were generated based on the Bray–Curtis distances generated by mothur. **(B)** PCoA plots based on weighted UniFrac distances of gut microbiome of oriental white storks. **(C)** PCoA plots based on unweighted UniFrac distances of gut microbiome of oriental white storks.

The relationship between the feces samples from two breeding conditions were represented by a dendrogram using Bray-Curtis distances (Figure 3A). This dendrogram indicated that the gut microbiota of the oriental white stork in Tianjin Zoo clustered together, while those in Tianjin Qilihai Wetland located on similar branches. To evaluate the difference in β-diversity of the 24 samples, PCoA was used to visualize similarities or dissimilarities between the Z and W groups (Figures 3B,C). Each symbol represents one gut microbiota on the PCoA plot, and most of the fecal samples in two groups were distinguishable by PCoA when considering the relative abundances of gut microbiomes. Congruent with the cluster analysis, bacterial communities of group Z clustered tightly and were separated from those of group W under both weighted and unweighted UniFrac metrics. The two groups tended to cluster separately from each other, indicating that each group had distinctive microbial populations. Using weighted UniFrac distance, bacterial communities of group Z were separated from those of group W along principal coordinate axis 1 (PC1) with the largest amount of variation (42.10%) (Figure 3B). Using unweighted UniFrac distance, bacterial communities of the 24 fecal samples explained the largest amount of variation (47.89%) along PC1 (Figure 3C). This clustering pattern was confirmed by an analysis of similarities (ANOSIM) and unweighted UniFrac showed a clearer separation of communities (ANOSIM, *R* = 0.71). Microbial composition between two groups at the phylum level (ANOSIM, *R* = 0.5621, *p* = 0.001) and genus level (ANOSIM, *R* = 0.6196, *p* = 0.001) were significantly different (Supplementary Figure 6).

Unique and shared bacterial taxa of the gut microbiota from two groups were analyzed. A Venn diagram demonstrated that 1,299 OTUs from the 24 samples were shared as core bacterial OTUs and the unique OTUs in each group were 256 (Z group) and 839 (W group), respectively (Supplementary Figure 7).

**FIGURE 4:**
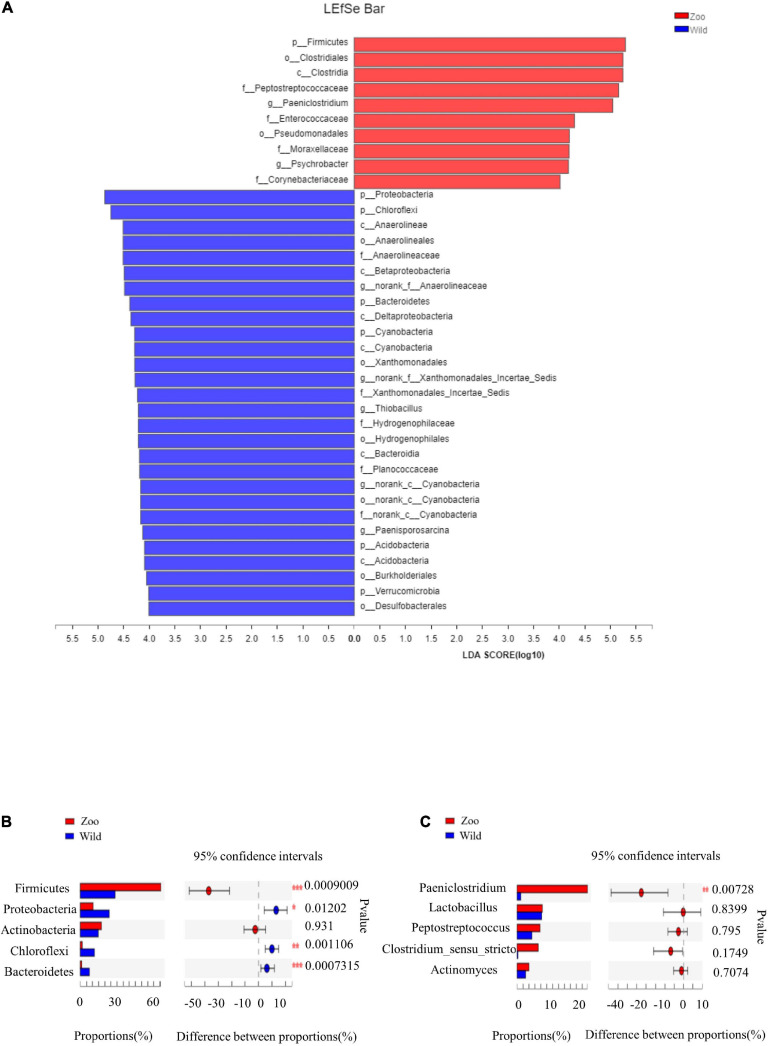
LEfSe analysis of gut microbiota and comparison of core gut microbiotas. **(A)** LEfSe analysis based on characterizing discriminative features of OTUs. **(B)** Top five abundant core phyla in two groups of oriental white storks. **(C)** Top five abundant core genera in two groups of oriental white storks.

There were some similarities in gut microbiota of oriental white storks from two groups. The top five abundant core phyla were Firmicutes, Proteobacteria, Actinobacteria, Chloroflexi, and Bacteroidetes, while the top five abundant core genera were *Paeniclostridium*, *Lactobacillus*, *Peptostreptococcus*, *Clostridium_sensu_stricto_1*, and *Actinomyces*. Next, LEfSe was used to identify OTUs differentially represented between oriental white storks from two groups (Figure 4A). LEfSe identified 10 and 28 taxa (LDA > 4.0) with discrepancies in relative abundance in the Z group and W group, respectively (Figure 4A).

The cladogram revealed that the core bacterial species in all 24 fecal samples were significantly different at all levels (Supplementary Figure 8). At the phylum level, the abundance of Firmicutes was significantly higher in the Z group than tin the W group, while the abundance of Bacteroidetes was significantly higher in the W group than in the Z group (*p* < 0.001) (Figure 4B). At the genus level, *Paeniclostridium* had a significantly higher abundance in the Z group than in the W group (*p* < 0.01) (Figure 4C).

**FIGURE 5:**
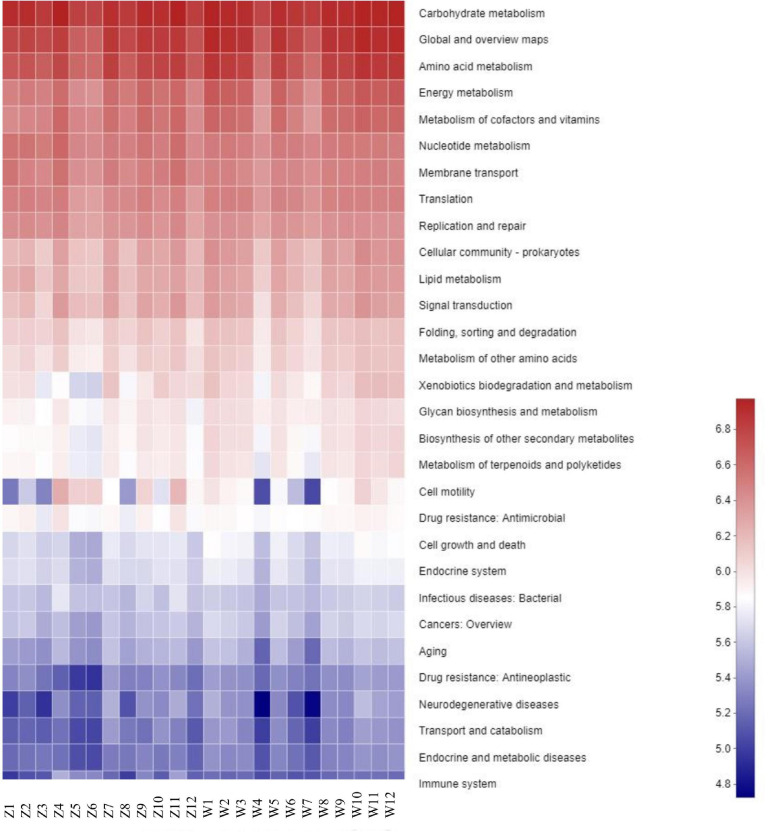
Heatmap of predictive KEGG functions in each sample.

A total of 296 KOs (KEGG Orthologs) and 42 KEGG pathways were mapped based on the high throughput sequencing, which were then classified into secondary KEGG pathways. The secondary KEGG pathways associated with the gut microbiotas included cell processes, metabolism, and genetic information processing, indicating that the differences in gut microbiota had important influences on the metabolism of *C. boyciana* (Figure 5).

## Discussion

This study characterized 24 fecal samples of oriental white storks living in two breeding condition, by sequencing of 16S rRNA gene amplicons using the Illumina MiSeq platform, analyzed the composition of the gut microbiota and compared the difference. The oriental white stork is one endangered waterbird species, and many conservation strategies have been brought up to protect *C. boyciana*, including establishment of suitable nature reserves (Zhou et al., 2013; Zheng et al., 2016; Tawa and Sagawa, 2020). However, there is limited knowledge of the gut microbiome of *C. boyciana* to efficiently support related conservation efforts. Therefore, to identify the gut microbiota associated with oriental white storks and differences in bacterial communities arising from captive and wild breeding groups, high-throughput 16S rRNA gene sequencing technology was employed to compare gut microbial compositions of oriental white storks from Tianjin Zoo and Tianjin Qilihai Wetland, respectively. This method allowed exploration of the microbiota composition and abundance without the need for cultivation (Caporaso et al., 2010; Gloor et al., 2010; Knutie and Gotanda, 2018).

### Microbiota Composition and Relative Abundance

Characterization of bacterial communities in the feces of oriental white storks provided evidence that the microbiota compositions were similar between the captivity and wild breeding groups, but abundances of the bacteria were significantly different between two groups. Gut microbiota of *C. boyciana* were predominantly composed of Firmicutes, Proteobacteria, and Actinobacteria at the phylum level, and Firmicutes was the most abundant phylum of Gram-positive bacteria in both Z group and W group. This microbiota composition was consistent with that of other wild bird species such as Canada goose (Lu et al., 2009), red-crowned cranes (Xie et al., 2016), Darwin’s finches (Michel et al., 2018), chinstrap penguins (Barbosa et al., 2016), northern bald ibis (Spergser et al., 2018), and kakapo (Waite et al., 2014), where members of the phylum Firmicutes dominated the microbiota of avian guts.

Members of the phylum Firmicutes play important roles in metabolism, digestion, and absorption of protein and other nutrients (Bernini et al., 2016; Berry, 2016). For example, Firmicutes are associated with breakdown of fatty acids, and Firmicutes produce more butyrate are which is considered a health-promoting molecule, since it can increase insulin sensitivity, regulate energy metabolism, and increase leptin gene expression. Bacteroidetes mainly produce acetate and propionate. Propionate could reduce the expression of enzymes involved in the *de novo* synthesis of fatty acids and Acetate could promote the secretion of insulin and ghrelin (Kong et al., 2013; Million et al., 2013; Lee et al., 2015). Although some studies speculated that the Firmicutes-to-Bacteroidetes (F/B) ratio may be related to obesity both in many animal models (Turnbaugh et al., 2006) and humans (Ley et al., 2006), the evidence suggesting an association between obesity and alterations of the Firmicutes/Bacteroidetes ratio is not convincing (Magne et al., 2020). In our study, the F/B ratio in Z group was higher than the ratio in W group, indicating that the gut microbiota of *C. boyciana* living in Tianjin Zoo could help the host use the nutrients more efficiently and collect calories from food since Firmicutes are more effective. It is difficult to obtain the physical indicators of oriental white storks living in Tianjin Qilihai Wetland, such as weight and health status, thus the relationship between the Firmicutes/Bacteroidetes ratio and obesity of two groups were still unknown.

*Paeniclostridium* was the most common genus in Z group, while *Lactobacillus* was the most common genus in W group and was also a major component of the gut microbiota in Z group. The abundance of *Lactobacillus* was relatively high in both Z group and W group (8.55% and 8.33%, respectively). In previous reports, *Lactobacillus* were represented in gut microbiota communities that had relatively high β-xylosidase and β-glucosidase levels (Ruggiero et al., 2009; Cabre et al., 2012). The similarity in relative abundance of *Lactobacillus* in both groups in the current study might be due to the formulation of the diet. The diet of oriental white storks is relatively large, and although the dietary structure is different in two groups, the amounts of sugar consumed in the different environments might be similar. Thus, it would be interesting to study the relationship between dietary structure with habitation and explore the functions of these bacteria in the metabolism pathways of *C. boyciana*.

### β-Diversity Analysis and Community Structures

The UniFrac method that focused on the phylogenetic relationships was developed by Lozupone and Knight (2005). Both weighted and unweighted UniFrac metrics are very useful for analyzing the differences and associations of microbial communities (Lozupone and Knight, 2005; Campbell et al., 2015). In this study, both weighted and unweighted UniFrac metrics suggested that each fecal sample harbors different microbial communities, and the majority of gut microbiotas were conserved and clustered together among different breeding conditions of oriental white storks (Z group and W group).

PICRUSt was used to analyze the microbial functions of oriental white storks. Gut microbial taxa of *C. boyciana* were associated with functions such as cell processes and metabolism. These data facilitate understanding of the relationship between gut microbial taxa and metabolism, as well as the influence of gut microbes on host health (Sommer and Backhed, 2013). However, as in other avian studies on gut microbiota, one limitation of the current study is the acquirement of DNA from each individual bird, as this affects the study of gut microbiota in relation to host genetics and physiology at the individual level (Liu et al., 2020).

Analysis of the 24 fecal samples demonstrated that the gut microbiota of oriental white storks was similar to those of red-crowned cranes and black-necked cranes at the phylum level, while Firmicutes was the predominant phylum (Xie et al., 2016; Wang et al., 2020). Comparison of the relative abundance of gut microbiota in *C. boyciana* between Z group and W group revealed that community structure and abundance of gut microbiota were relatively stable at the phylum level, but were variable and complex at the genus level. There was a significant difference in the richness and diversity of microbial composition, and host intestinal microbiota may be related to diet (Turnbaugh et al., 2009), geography, and seasonal changes (Mueller et al., 2006). The differences between Z group and W group were living conditions and dietary conditions. Oriental white storks inhabiting Tianjin Qilihai Wetland required higher energy and had a more diverse diet, so W group had a greater abundance of gut microbiota than Z group. This study suggests that biogeographical and dietary factors may contribute to the community structure of the gut microbiota of *C. boyciana* since the metabolism and energy harvest needs were different between two groups. This is consistent with previous related avian findings that community structure and abundance of the gut microbiota are mainly affected by dietary conditions (Wang et al., 2020). The difference on diet patterns may contribute to the differences of fecal microbiota on two groups, and more work is needed to understand the functions of diet patterns and living condition on fecal microbiota. So next, whether living condition changes in the fecal microbiota will be investigated using a combination of metagenomics and metabolomics. Furthermore, since the different microbiota could have different effects on the digestion ability of carbohydrates, protein, and cellulose, the differences in feed composition will have important influences on the changes in host gut microbiota. This may affect nutrient utilization and could cause gastrointestinal diseases in oriental white storks. Hence, studies on the gut microbiota of *C. boyciana* will facilitate understanding of the specific functions of different bacteria related to digestion and absorption in the host, and could help improve conservation for threatened species both in artificial breeding groups and in wild groups.

## Conclusion

This study characterized the gut microbiota of oriental white storks under different breeding conditions using 16S rRNA gene sequencing analysis. Microbiota compositions were similar between Z group and W group, but the abundances of bacteria differed. The genetic factors were the same, therefore it is hypothesized that the differences are mainly related to the environment and diet. Most gut microbiotas were clustered, respectively, between the different breeding conditions, and the gut microbiota were related to host metabolic pathways. This study provides insights into the composition and differences in gut microbiota of oriental white storks living in two breeding conditions. The findings will facilitate our further understanding of the relationship between biogeography, diet structure, and species diversity of the gut microbiota in oriental white storks, and thus help for the integrative conservation of this endangered species.

## Data Availability Statement

The datasets presented in this study can be found in online repositories. The names of the repository/repositories and accession number(s) can be found in the article/Supplementary Material.

## Author Contributions

D-PZ, HW, and Q-HZ conceived and designed the study and wrote the manuscript. F-TW collected the samples. HW and F-TW conducted the experiments. D-PZ, HW, and F-TW analyzed the data. All authors contributed to the article and approved the submitted version.

## Conflict of Interest

The authors declare that the research was conducted in the absence of any commercial or financial relationships that could be construed as a potential conflict of interest.

## Footnotes

1 http://rdp.cme.msu.edu/

2 https://www.arb-silva.de
