# Supplementary figures and images for: Comparative Analysis of Gut Microbiota in Captive and Wild Oriental White Storks: Implications for Conservation Biology
Source: Front Microbiol. 2021 Mar 25;12:649466. doi: 10.3389/fmicb.2021.649466 (PMC8027120; doi:10.3389/fmicb.2021.649466)

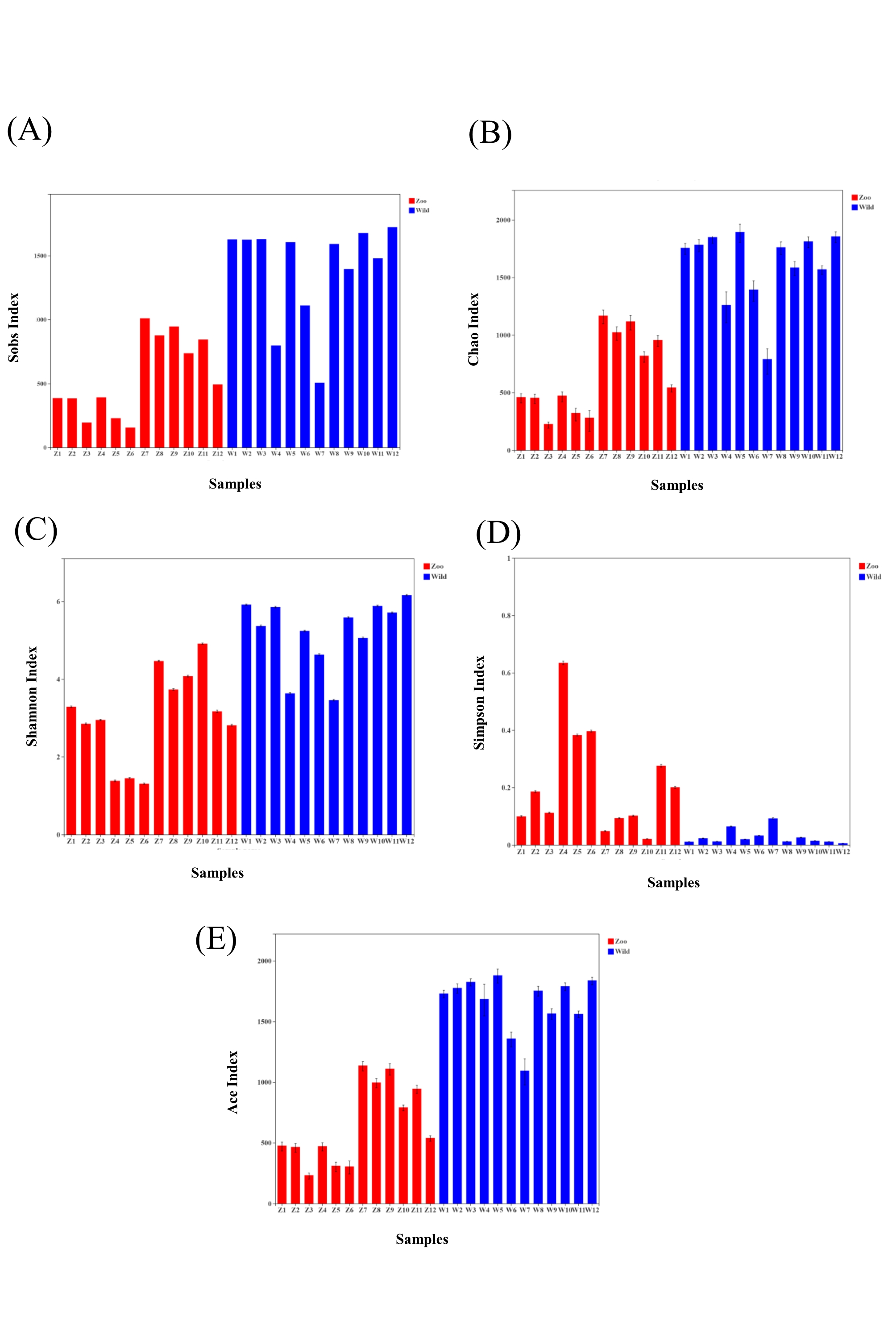

Supplement: Supplementary file 1 [file Image_1.JPEG]

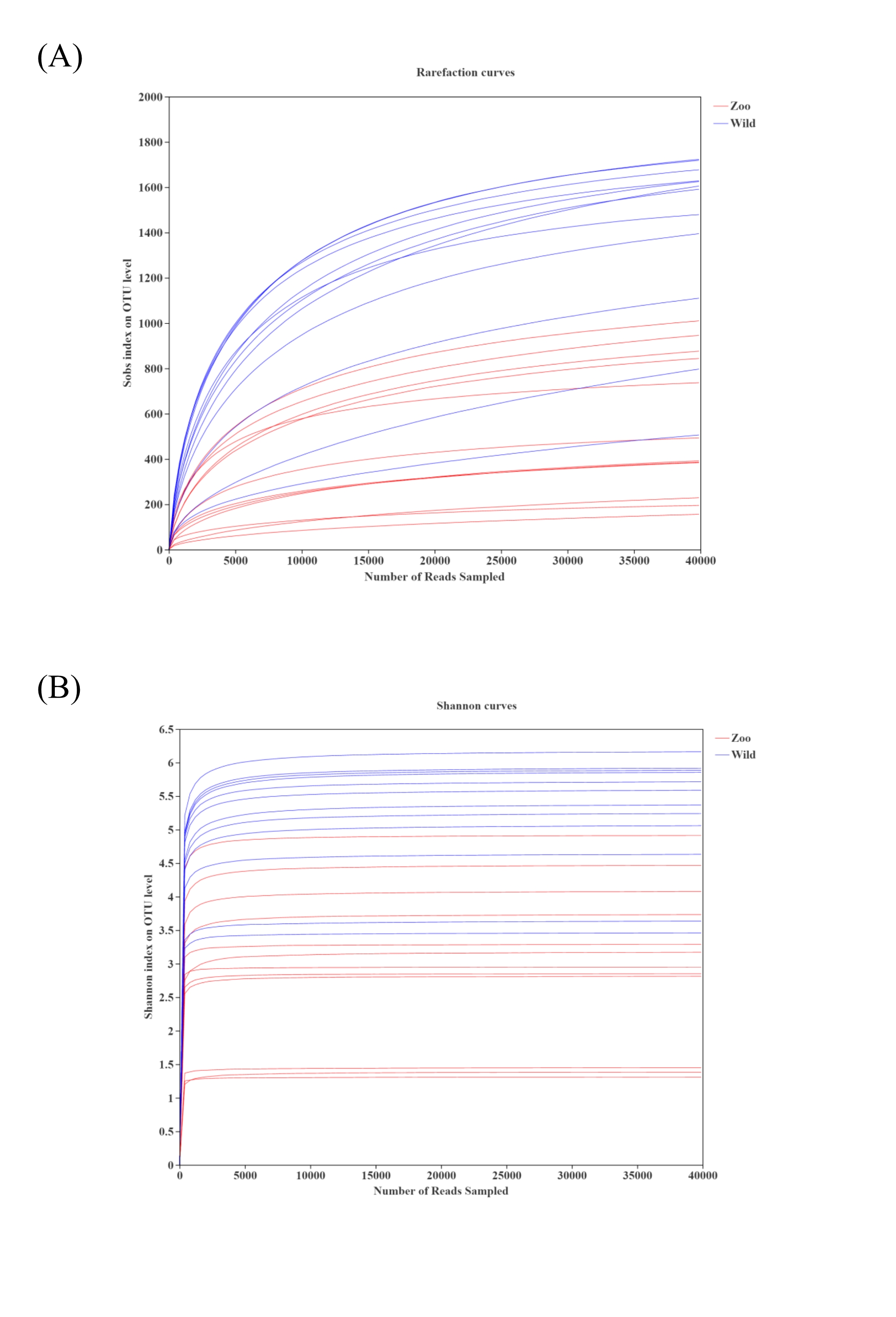

Supplement: Supplementary file 2 [file Image_2.JPEG]

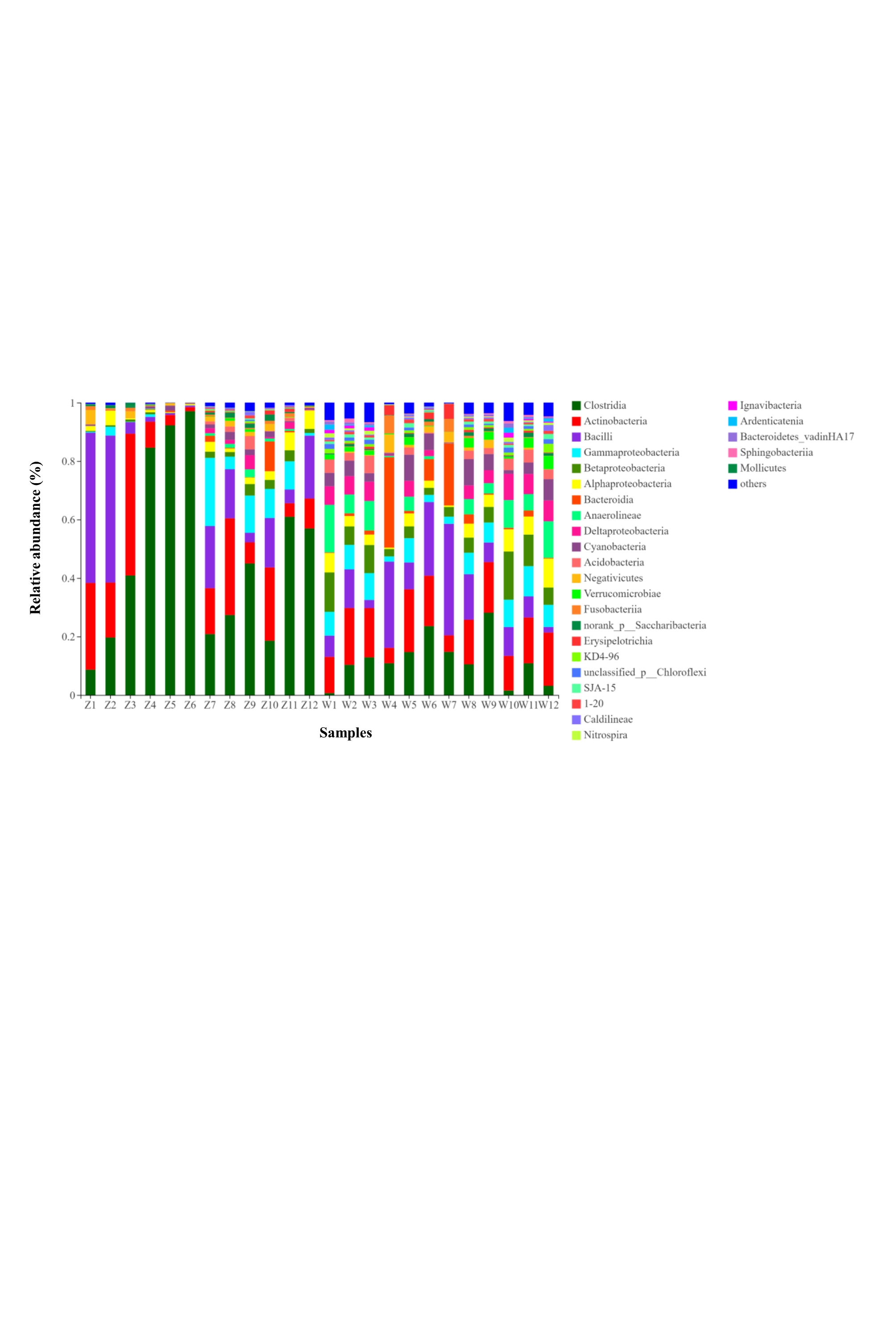

Supplement: Supplementary file 3 [file Image_3.JPEG]

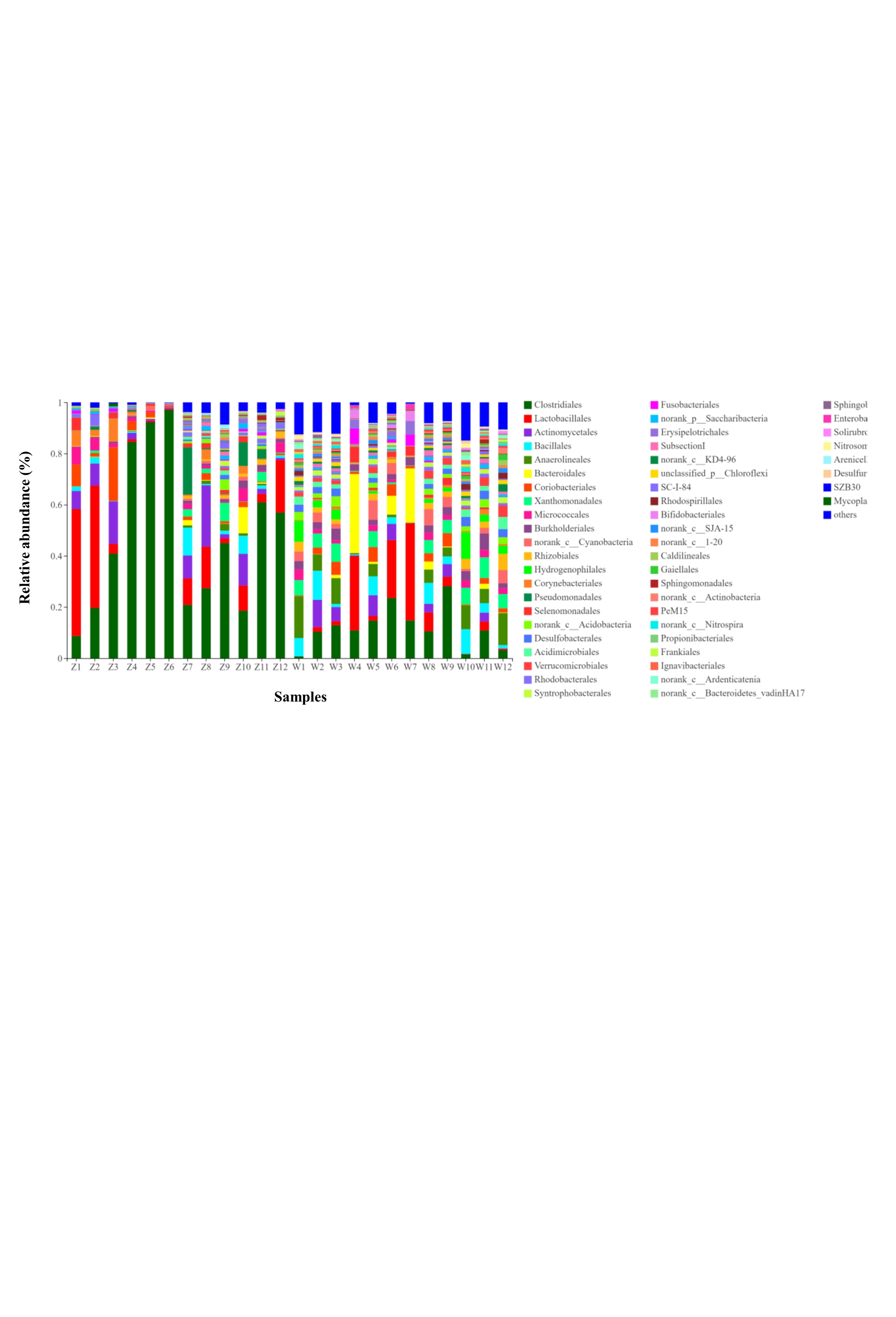

Supplement: Supplementary file 4 [file Image_4.JPEG]

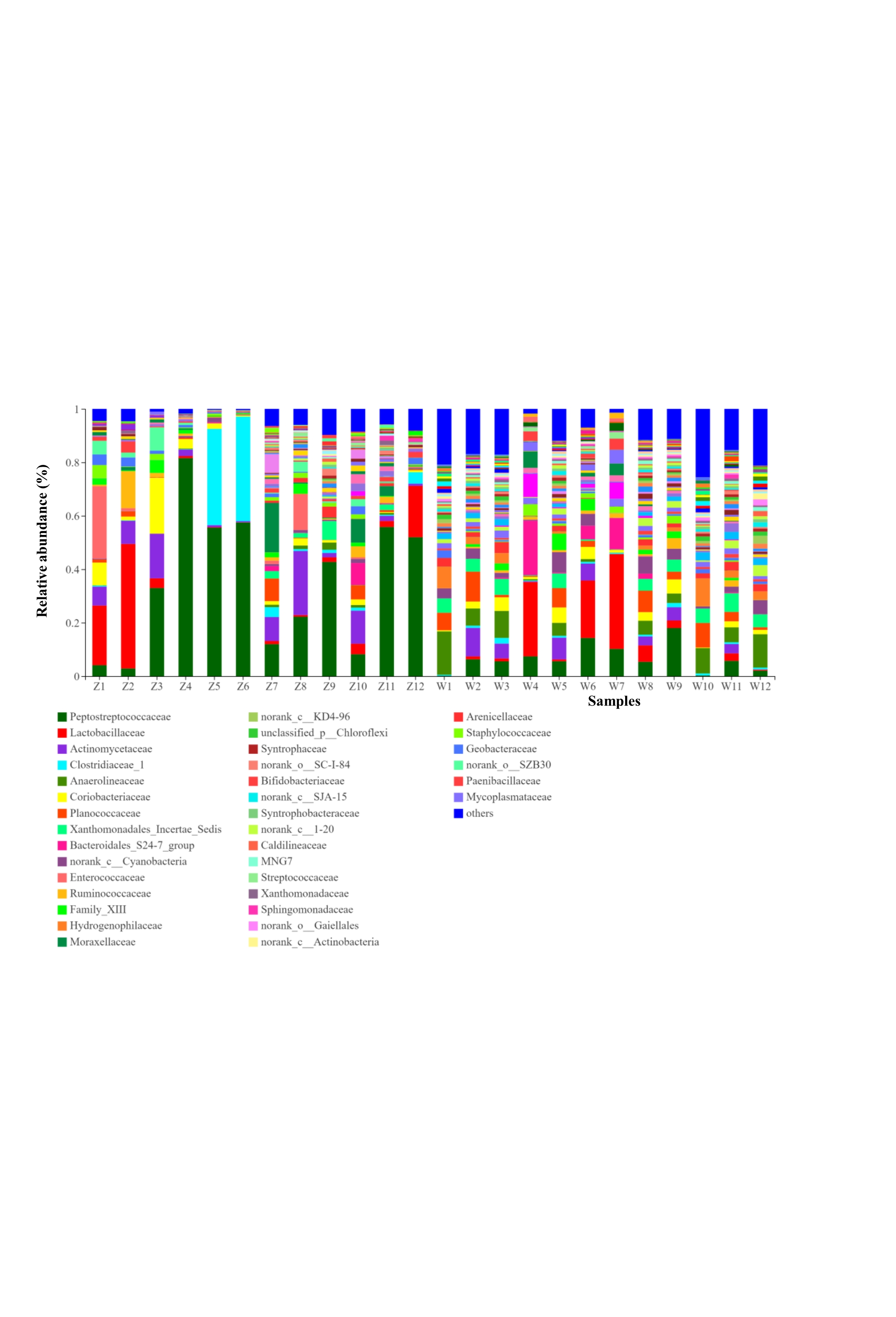

Supplement: Supplementary file 5 [file Image_5.JPEG]

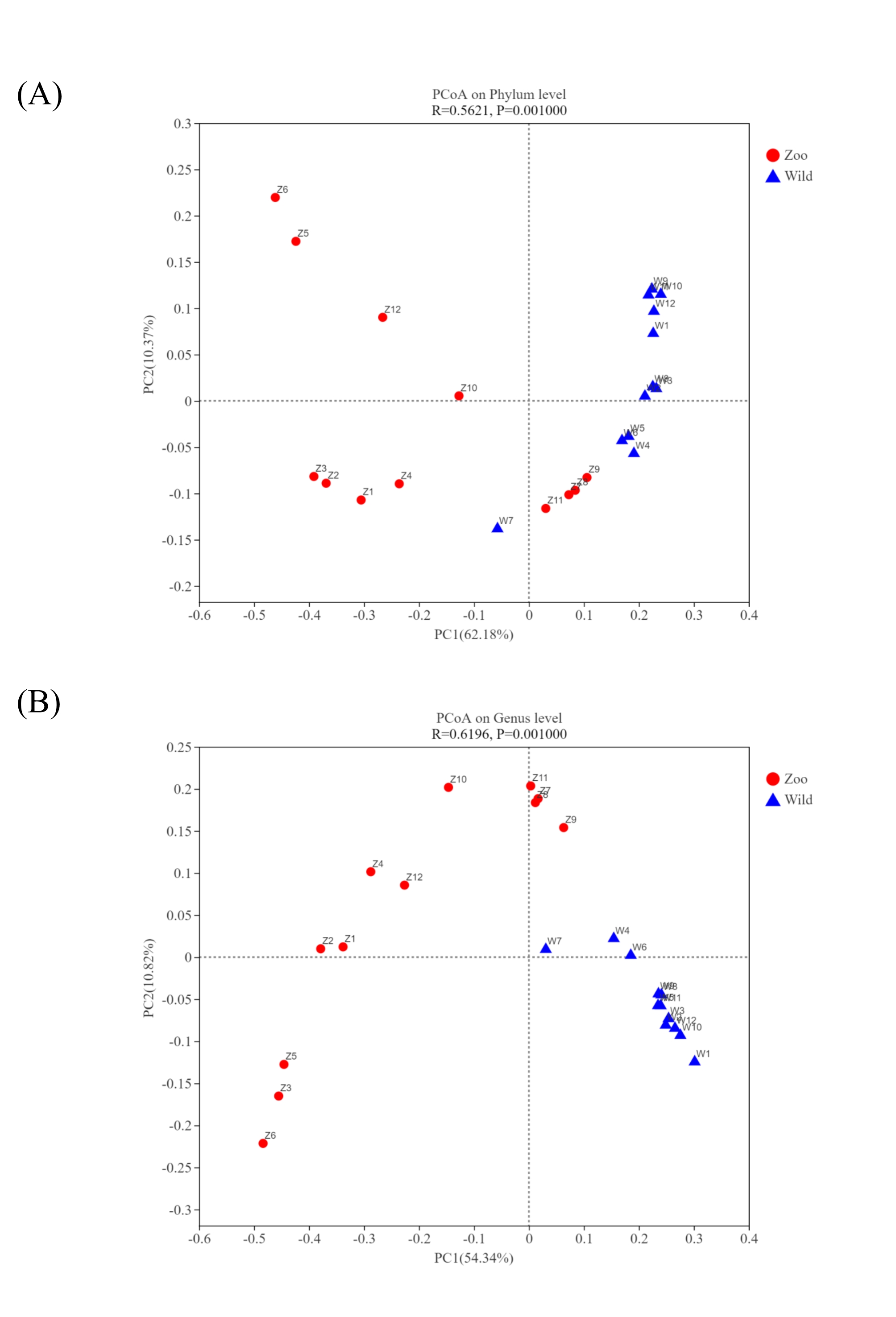

Supplement: Supplementary file 6 [file Image_6.JPEG]

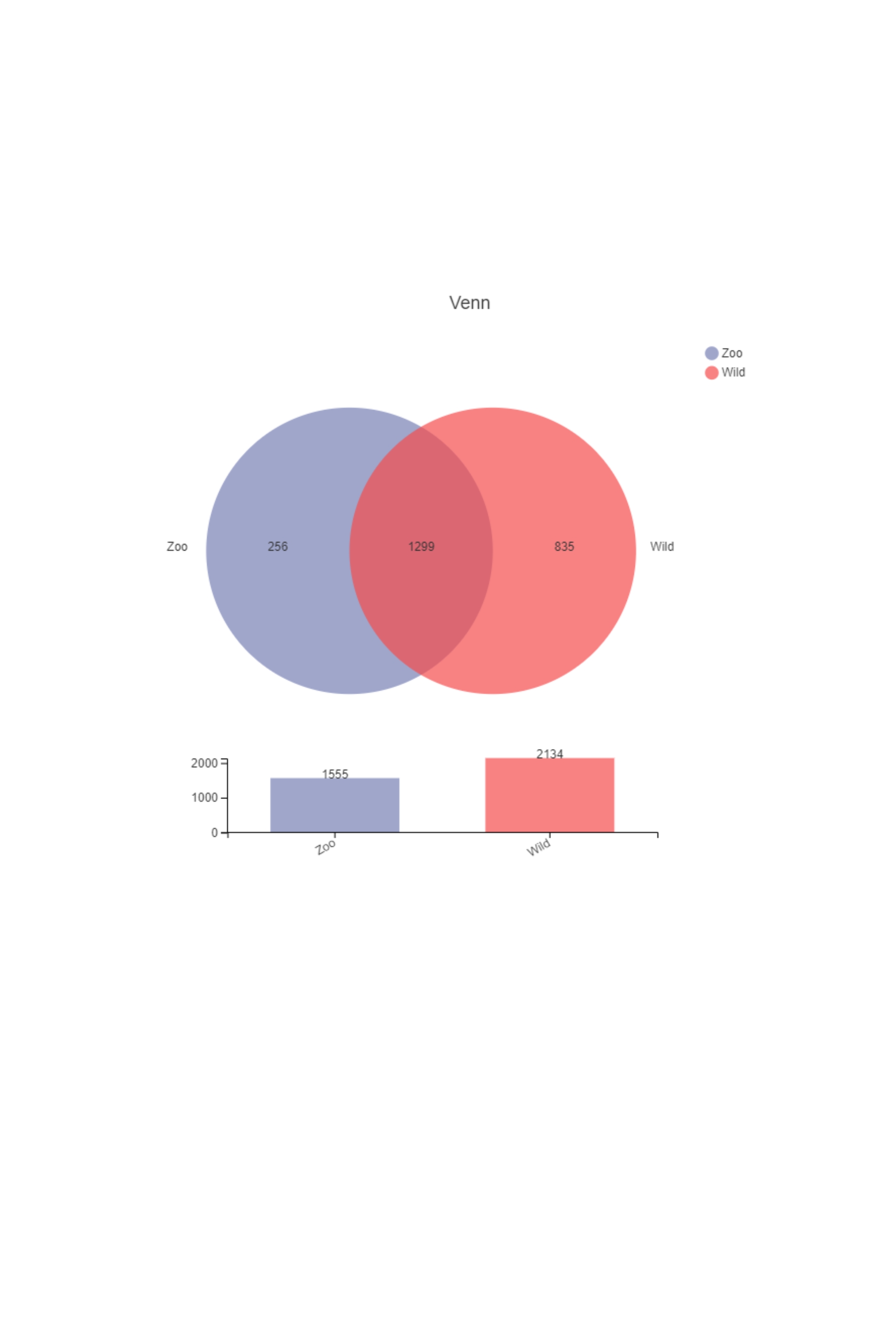

Supplement: Supplementary file 7 [file Image_7.JPEG]

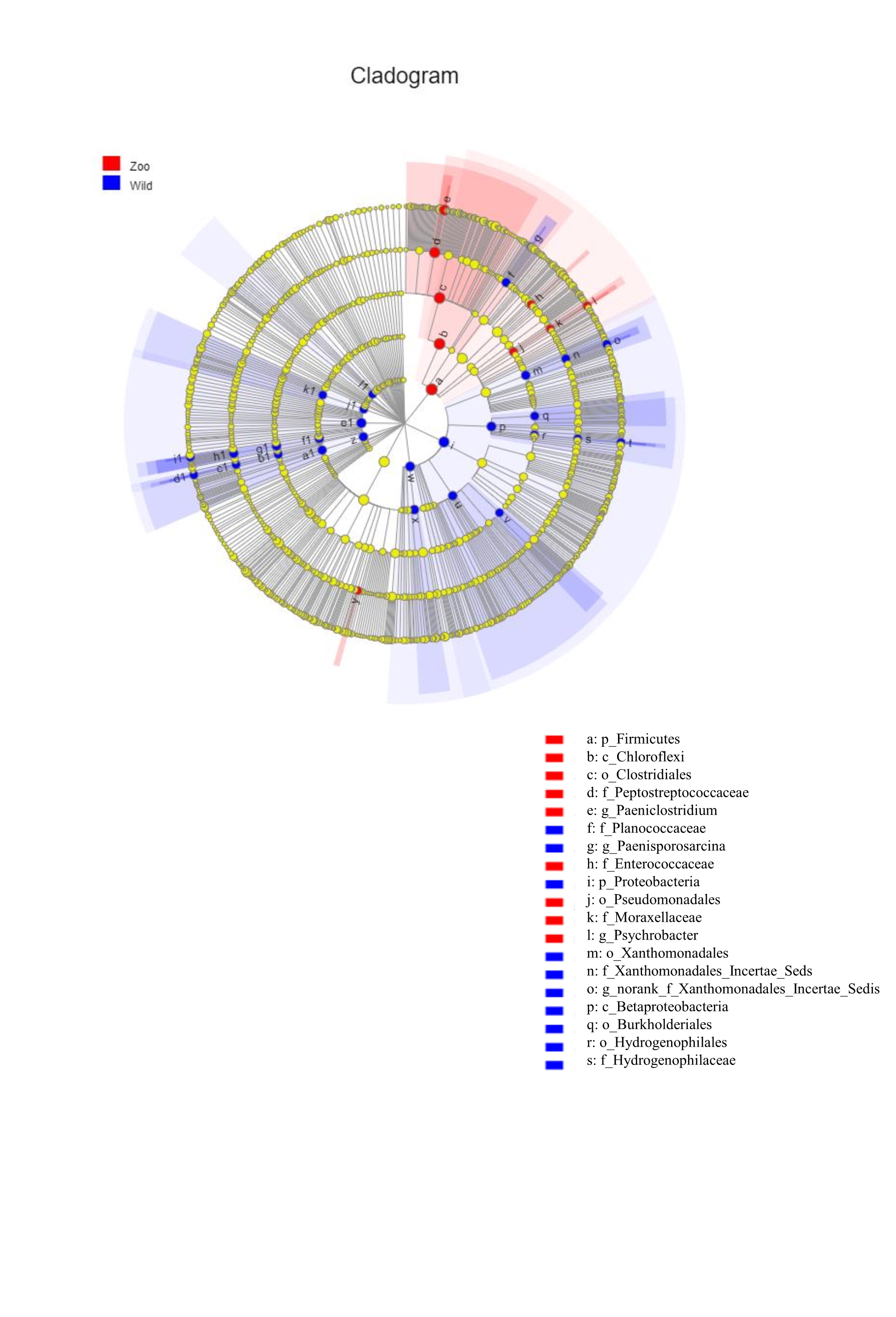

Supplement: Supplementary file 8 [file Image_8.JPEG]
