# Supplementary material for: Comparative Analysis of Gut Microbiota in Captive and Wild Oriental White Storks: Implications for Conservation Biology
Source: Front Microbiol. 2021 Mar 25;12:649466. doi: 10.3389/fmicb.2021.649466 (PMC8027120; doi:10.3389/fmicb.2021.649466)
Supplement: Supplementary file 9 [file Table_1.DOCX]

Table S1. The total number of raw reads, base pairs, the mean length of the reads and number of bacterial taxonomic units

| **Sample Information** | **Seq_num** | **Base_num** | **Mean_length** | **Phyla** | **Class** | **Order** | **Family** | **Genera** | **Species** | **OTU** |
| --- | --- | --- | --- | --- | --- | --- | --- | --- | --- | --- |
| Z1 | 70383 | 29619286 | 420.8301 | 7 | 11 | 22 | 41 | 58 | 68 | 78 |
| Z2 | 47926 | 20108391 | 419.5717 | 7 | 12 | 25 | 43 | 65 | 78 | 96 |
| Z3 | 53002 | 21756213 | 410.4791 | 6 | 10 | 15 | 24 | 37 | 44 | 54 |
| Z4 | 51957 | 21044137 | 405.0299 | 6 | 13 | 24 | 34 | 47 | 54 | 62 |
| Z5 | 46161 | 18661981 | 404.2803 | 5 | 7 | 9 | 13 | 19 | 23 | 27 |
| Z6 | 57459 | 23202813 | 403.8151 | 4 | 6 | 9 | 13 | 16 | 20 | 22 |
| Z7 | 42700 | 17897453 | 419.1441 | 10 | 24 | 50 | 76 | 125 | 154 | 192 |
| Z8 | 45309 | 18857741 | 416.203 | 11 | 24 | 47 | 63 | 89 | 110 | 153 |
| Z9 | 53407 | 22053370 | 412.9303 | 15 | 27 | 56 | 72 | 97 | 134 | 195 |
| Z10 | 47528 | 19845875 | 417.5618 | 10 | 21 | 40 | 71 | 132 | 179 | 237 |
| Z11 | 52498 | 21538789 | 410.2783 | 11 | 22 | 45 | 69 | 112 | 140 | 164 |
| Z12 | 52871 | 21715875 | 410.7332 | 7 | 14 | 31 | 59 | 90 | 103 | 115 |
| W1 | 62080 | 25882420 | 416.9204 | 14 | 34 | 73 | 107 | 145 | 228 | 377 |
| W2 | 45143 | 18860860 | 417.8025 | 21 | 43 | 78 | 113 | 155 | 207 | 284 |
| W3 | 55742 | 23226082 | 416.6711 | 21 | 42 | 82 | 120 | 161 | 239 | 341 |
| W4 | 56282 | 23740408 | 421.8117 | 7 | 13 | 24 | 36 | 63 | 88 | 104 |
| W5 | 64088 | 26642656 | 415.7199 | 17 | 38 | 73 | 102 | 140 | 183 | 250 |
| W6 | 48989 | 20428451 | 417.0008 | 11 | 24 | 51 | 78 | 115 | 152 | 186 |
| W7 | 45156 | 19056753 | 422.0204 | 5 | 11 | 17 | 29 | 64 | 89 | 106 |
| W8 | 46650 | 19451236 | 416.9611 | 17 | 35 | 71 | 103 | 149 | 207 | 282 |
| W9 | 45418 | 18814827 | 414.2593 | 17 | 35 | 68 | 97 | 135 | 178 | 234 |
| W10 | 58817 | 24640596 | 418.9366 | 19 | 41 | 84 | 129 | 179 | 266 | 399 |
| W11 | 50511 | 21102860 | 417.7874 | 17 | 38 | 81 | 125 | 177 | 240 | 314 |
| W12 | 63262 | 26209729 | 414.3045 | 12 | 35 | 74 | 112 | 161 | 235 | 377 |

Table S2. Mean relative abundance of the 10 most abundant phyla in Tianjin Zoo and Qilihai Wetland

| **Sample group** | **Top ten abundant phyla (%)** |
| --- | --- |
| Tianjin Zoo (Z group) | Firmicutes (65.59) |
|  | Actinobacteria (17.23) |
|  | Proteobacteria (10.48) |
|  | Chloroflexi (1.61) |
|  | Bacteroidetes (1.22) |
|  | Cyanobacteria (1.02) |
|  | Saccharibacteria (0.78) |
|  | Acidobacteria (0.74) |
|  | Fusobacteria (0.53) |
|  | Verrucomicrobia (0.27) |
| Qilihai Wetland (W group) | Firmicutes (28.43) |
|  | Proteobacteria (23.58) |
|  | Actinobacteria (14.71) |
|  | Chloroflexi (11.57) |
|  | Bacteroidetes (7.42) |
|  | Cyanobacteria (4.59) |
|  | Acidobacteria (2.75) |
|  | Verrucomicrobia (2.15) |
|  | Fusobacteria (1.15) |
|  | Saccharibacteria (0.72) |
